# FosA8-producing *E. coli* ST131: clinical cases in Italy, February 2023

**DOI:** 10.2807/1560-7917.ES.2024.29.21.2400276

**Published:** 2024-05-23

**Authors:** Katerina Chudejova, Maria Sofia Caltagirone, Vittoria Mattioni Marchetti, Antonella Rezzani, Antonella Navarra, Ibrahim Bitar

**Affiliations:** 1 Department of Microbiology, Faculty of Medicine, University Hospital in Pilsen, Charles University, Pilsen, Czechia; 2 Biomedical Center, Faculty of Medicine, Charles University, Pilsen, Czechia; 3 Microbiology Unit, IRCCS Istituti Clinici Scientifici Maugeri, Pavia, Italy; 4 Scienze Clinico, Chirurgiche, Diagnostiche, Pediatriche Department, Microbiology Unit, University of Pavia, Pavia, Italy; 5 Specialization School of Microbiology and Virology, University of Pavia, Pavia, Italy

**Keywords:** Fosfomycin, *E. coli* ST131, FosA8, Fosfomycin resistance,

## Abstract

Fosfomycin-resistant FosA8-producing *Enterobacterales* are uncommon strains with extremely low incidence in Europe, based on only three reports in the literature. We detected FosA8-producing *Escherichia coli* ST131 in clinical isolates from two patients admitted in February 2023 to a rehabilitation unit in Italy. The occurrence of rare *fosA*-like genes in the high-risk clone ST131 is of clinical relevance. The dissemination of FosA-producing *E. coli,* although still at low levels, should be continuously monitored.

Fosfomycin (FOS) is an old bactericidal, broad-spectrum antibiotic recently revived in clinical practice for treatment of severe infections, including those caused by multidrug-resistant organisms [1,2]. Resistance to FOS can develop through acquisition of antimicrobial resistance genes, such as the *fosA* family. Despite the need for comprehensive surveillance, characterisation of FOS resistance in Europe remains scarce. Here, we characterise, to the best of our knowledge, the first two FOS-resistant ST131 *Escherichia coli* strains identified in a clinical setting in Italy using whole genome sequencing (WGS).

## Detection of the resistant strains 

The Istituti Clinici Scientifici (ICS) Maugeri in Turin, Italy is an 80-bed long-term acute care rehabilitation facility. Two patients (P13 and P15) were admitted 2 days apart in mid-February 2023 to the neuromotor rehabilitation unit of the ICS Maugeri. Both patients developed typical symptoms of urinary tract infection, and urine samples were collected and sent for culture, before empirical antibiotic therapy was started. *Escherichia coli* strains, designated EC13_PV and EC15_PV, were isolated from urine samples from the patients in mid-February. The two strains were isolated as part of a 1-year surveillance program on FOS-resistant *Enterobacterales* among ICS Maugeri’s institutes. 

Both patients developed clinical symptoms 3–4 days after admission. Patients were treated empirically with Augmentin. After treatment, urine culture results negative. The patients did not share a room, but they attended the same shared spots in the rehabilitation unit. 

## Antimicrobial and phenotypic profiles

Species identification and susceptibility testing were carried out using the MicroScan WalkAway System (Beckman Coulter). The results were interpreted according to European Committee on Antimicrobial Susceptibility Testing (EUCAST) 2023 criteria [3]. The two *E. coli* strains shared the same multidrug-resistant profile, retaining susceptibility only to aminoglycosides, carbapenems, nitrofurantoin and piperacillin/tazobactam and showing resistance to amoxicillin-clavulanic acid, cephalosporins, fluoroquinolones, FOS and trimethoprim/sulfamethoxazole (Table 1). 

**Table 1:** Antimicrobial susceptibility of *E. coli* isolates from two patients, Turin, Italy, February 2023

Strain	AK	AMC	CAZ	CTX	FEP	CIP	GN	FOS	ETP	MER	IMI	NT	PTZ	TO	SMX
EC13_PV	≤ 8 S	32 R	32 R	> 32 R	> 8 R	> 1 R	≤ 2 S	> 32 R	≤ 0.12 S	≤ 0.12 S	≤ 1 S	≤ 64 S	≤ 4 S	> 4R	> 4/76 R
EC15_PV	≤ 8 S	32 R	32 R	> 32 R	> 8 R	> 1 R	≤ 2 S	> 32 R	≤ 0.12 S	≤ 0.12 S	≤ 1 S	≤ 64 S	≤ 4 S	> 4R	> 4/76 R

AK: amikacin; AMC: amoxicillin/clavulanic acid; CAZ: ceftazidime; CIP: ciprofloxacin; CTX: cefotaxime; *E*: *Escherichia*; ETP: ertapenem; FEP: cefepime; FOS: fosfomycin; GN: gentamycin; IMI: imipenem; MER: meropenem; MIC: minimum inhibitory concentration; NT: nitrofurantoin; PTZ: piperacillin/tazobactam; R: resistant; S: susceptible; SMX: trimethoprim/sulfamethoxazole; TO: tobramycin.

Interpretation was assessed according to EUCAST clinical breakpoints 2023 [3]. Results are expressed in mg/L.

The FOS minimum inhibitory concentrations (MIC) were assessed by the EUCAST reference agar-dilution method (ADM) (Liofilchem Diagnostics) and the presence of plasmid-encoded FosA enzymes by using the disk potentiation testing with sodium phosphonoformate (PPF) [4]. The resistance to FOS was confirmed for both strains by ADM (MIC =  > 128 mg/L) and the PPF test highlighted the production of FosA-like enzymes.

## Molecular characterisation

Whole genome sequencing on the Illumina NovaSeq platform was conducted on both strains. The reads obtained were de novo assembled with Shovill while the reconstruction of the resistome, plasmidome and virulome of the isolates using ResFinder 4.5.0 , PlasmidFinder 2.1 and the Virulence Factors Database (VFDB) via ABRicate. The multilocus sequence typing (MLST) profiles were assigned according to the Achtman scheme on Enterobase, while plasmid multilocus sequence typing (pMLST) was investigated through pMLST 2.0 [5]. 

The WGS (coverage 70X for both) revealed the presence of the *fosA8* variant in both the isolates, together with genes involved in aminoglycoside (*aadA5*, *aac(6')-Ib-cr*), chloramphenicol (*catB3*), trimethoprim (*dfrA17*), macrolides (*mph(A)*), sulfonamide (*sul1*) and beta-lactam (*bla*CTX-M15, *bla*OXA-1) resistance (Table 2). Moreover, strains EC13_PV and EC15_PV belonged to the ST131 clade C, serotype O25b:H4, and shared same plasmid content, consisting of Col440II, ColRNAI, IncFIA-IncFIB-IncFII (pMLST IncF F31:A4:B1) and IncN (pMLST IncN unknown) (Table 2).

**Table 2:** Metadata and genomic characterisation of FosA8-producing ST131 *E. coli* isolates from two patients, Turin, Italy, February 2023

**Strain**	**Isolation date**	**Specimen**	**Serotype**	**FimH**	**MLST**	**Clade**	**Resistome**	**Plasmidome**	**pMLST**	**Accession number**
EC13_PV	Feb 2023	Urine	O25b:H4	H30	131	C	*aadA5*, *aac(6')-Ib-cr*, *bla*CTX-M-15, *bla*OXA-1, *catB3*, *dfrA17*, *fosA8*, *mph(A)*, *sul1*	Col440II, ColRNAI, IncFIA, IncFIB, IncFII, IncN	IncF F31:A4:B1, IncN unknown	JBAMKF000000000
EC15_PV	Feb 2023	Urine	O25b:H4	H30	131	C	*aadA5*, *aac(6')-Ib-cr*, *bla*CTX-M-15, *bla*OXA-1, *catB3*, *dfrA17*, *fosA8*, *mph(A)*, *sul1*	Col440II, ColRNAI, IncFIA, IncFIB, IncFII, IncN	IncF F31:A4:B1, IncN unknown	JBAMKG000000000

*E*.: *Escherichia*; MLST: multilocus sequence typing.

The two patients were admitted 2 days apart, and both developed symptoms 3-4 days after admission.

**Figure 1:**
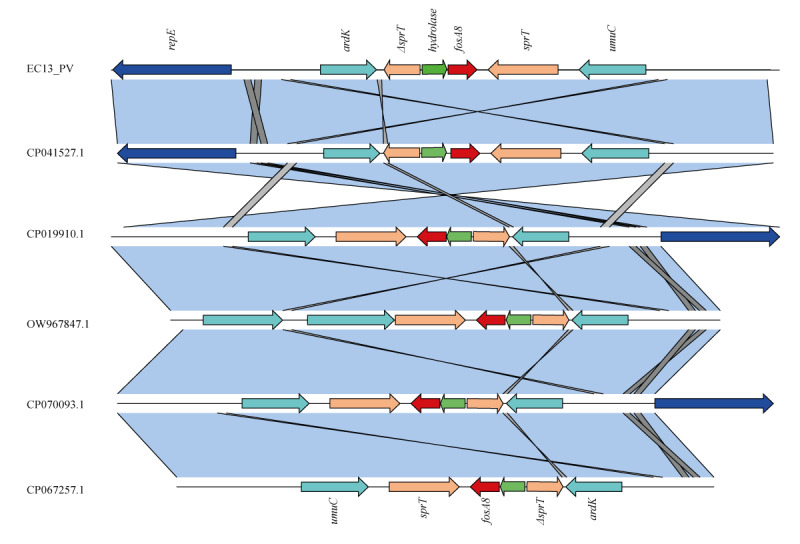
Linear visualisation of the *fosA8* genetic environment of patient EC13_PV, Turin, Italy, February 2023

The *fosA8* variant was in both cases located on the IncN plasmid, highly transferable by conjugation. The *fosA8* was inserted in a genomic environment of 1,590 bp, flanked by one copy of the *sprT* gene and one deleted *sprT* gene (*ΔsprT*), as reported elsewhere [6]. This genomic composition is shared at high level (ID > 99.00% and query = 100.00%) with the available genomes on National Center of Biotechnology Information (NCBI) belonging to different species: CP041527.1 (an *E. coli* collected in 2013 from the blood sample of a patient in the United States (US)), CP019910.1 (*E. coli* from a patient in the US collected in 2015), OW967847.1 (*Klebsiella pneumoniae* collected in 2021 from Spanish patient), CP070093.1 (*K. pneumoniae* from clinical sample in the US) and CP067257.1 (*K. pneumoniae* collected from food in Switzerland in 2014) (Figure 1). These results suggest a conserved nature of the *fosA8*-surrounding environment and an interspecies transferability of *fosA8*-harboring plasmids.

The figure was generated using EasyFig [13]. The arrows represent the annotated genes: genes for replication (blue); *sprT* gene (orange); genes for hydrolase (green); *fosA8* antimicrobial resistant genes (red); other genes (turquoise).

**Figure 2:**
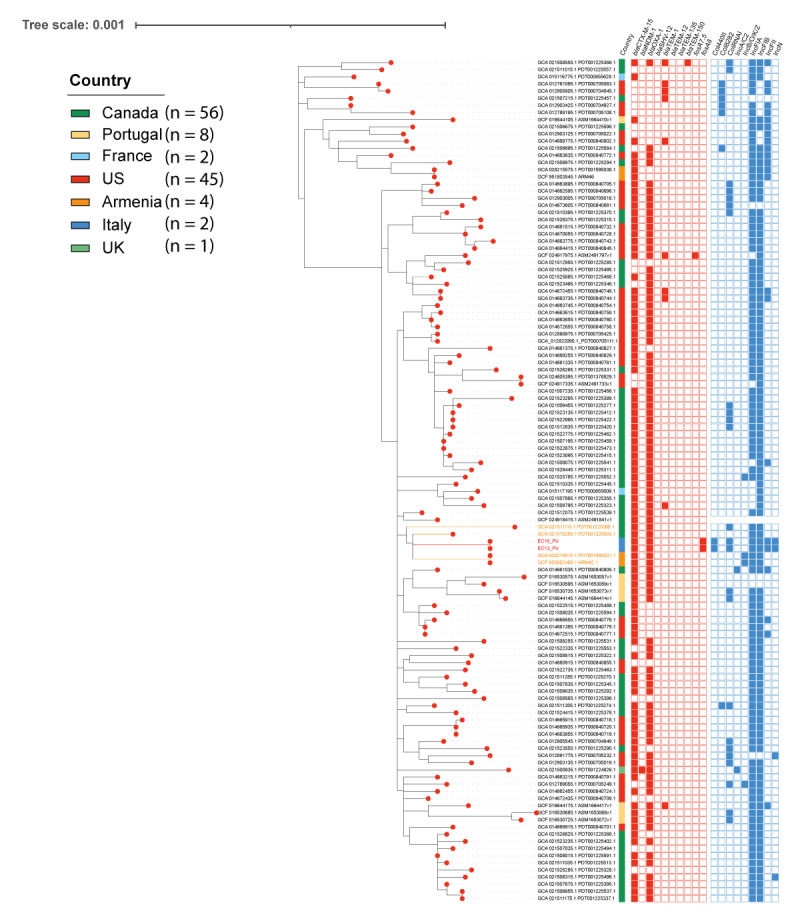
Maximum-likelihood phylogeny of *E. coli* ST131 strains inferred on coreSNPs, Turin, Italy, February 2023 (n = 2) and worldwide (n = 116)

Single nucleotide polymorphism (SNP)-based maximum-likelihood phylogeny was inferred using Parsnp v2.0.3 on the two *E. coli* strains and the 784 genomes belonging to ST131 O25b:H4 available in NCBI. The SNP-based approach inserted the two studied *E. coli* in a unique wide node including 116 genomes, all belonging to the same FastBaps cluster (Figure 2). EC13_PV and EC15_PV clustered together, showing a clonal relationship and revealing only one SNP difference (G78A). The two *E. coli* shared genetic relatedness with two *E. coli* (GCA_021511115.1; GCA_021510255.1) (SNPs range: 232–264) collected in 2016 in Canada from cases with bloodstream infection. Moreover, the two *E. coli* strains showed similar relatedness with two additional *E. coli* (GCA_028215515; GCF_955652485.1) (SNPs range: 276–317) collected in 2019 in Armenia from two cases with urinary tract infection (Figure 2).

*E*.: *Escherichia*; SNP: single nucleotide polymorphism; UK: United Kingdom; US: United States.

The phylogenetic tree was visualised using iTOL v6, including the two FosA8-producing *E. coli* ST131 (red labels). Labelled with orange text are the four genomes that are closely clustered with EC13_PV and EC15_PV. The presence/absence matrix shows the pattern of resistance determinants (red squares) and the plasmid content (blue squares).

**Figure 3:**
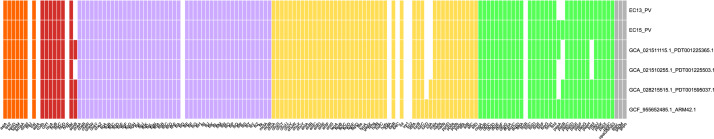
Heatmap representation of the virulence genes in *E. coli* strains from two patients, Turin, Italy, February 2023 (n = 2) and strains retrieved from NCBI (n = 4)

The two FosA8-producing *E. coli* and the four closely related strains showed a conserved virulence content, including genes for iron uptake systems (*sitABCD*, *chuA*, *fyuA* and *malX*), for invasion (*aslA*, *iss2*, *kpsDM*, *ompA*, *traT*), for chemotaxis (*che* locus, *flg* locus, *fli* locus, *flk*, *motAB*) and for adhesion (*csg*, *ecp* and *fim* loci, *pap*) (Figure 3). Moreover, all the six clustered strains shared the presence of toxin genes, such as *cnf1*, encoding the cytotoxic necrotising factor 1, *hly*ABCD, expressing haemolysins (A-D), and *sat*, the gene for the secreted autotransporter toxin (Figure 3).

*E*.: *Escherichia*; NCBI: National Center of Biotechnology Information.

Patient strains EC13_PV, EC15_PV are compared to strains GCA_021511115.1, GCA_021510255.1, GCA_028215515.1 and GCF_955652485.1, available at NCBI.

Colours describe virulence genes involved in invasion (orange), toxins (red), chemotaxis (lilac), metabolism (yellow), adhesion (green) and others (grey).

## Discussion

Fosfomycin resistance is an increasing threat among Enterobacterales, affecting the use of FOS in the treatment of severe infections. Given the lack of rapid diagnostic approaches for FOS MICs, evaluation and surveillance of FOS-resistant strains in clinical practice is challenging [2]. The use of commercial kits for ADM is difficult to add into routine practice because of the high cost, while development of in-house alternatives is too time-consuming. As a first step, the PPF test for *E. coli* strains can be used to confirm FOS categorisation (susceptible/resistant) and evaluate the production of FosA enzymes. The use of commercial ADM for high-risk pathogens with suspected FOS resistance could provide some clarity to the current picture of FOS resistance among Enterobacterales and could be a starting point for future surveillance programs.

Based on our data, we speculate nosocomial transmission. However, the absence of other similar cases within the rehabilitation unit, the lack of environmental samplings from the same clinical setting and the lack of community surveillance on these strains limit our speculation. For this reason, the introduction of surveillance for FosA enzyme circulation among Enterobacterales is essential to understanding the epidemiology of FOS resistance.

*E. coli* ST131 is known as a successful high-risk virulent clone, often causing urinary tract infections. ST131, particularly clade C, is associated with the dissemination of extended-spectrum beta-lactamases (ESBLs), as CTX-M-15 type, and with fluoroquinolone resistance [7]. The most common subtypes of *fosA*-like genes detected worldwide are *fosA3*, *fosA7* and *fosA4*, while other *fosA*-like variants including *fosA8* are rarely reported [2]. The occurrence of *fosA*-like genes in *E. coli* ST131 is rarely reported in literature, with sporadic cases in Turkey and China [8,9]. However, despite this low incidence, the presence of acquired *fosA*-like genes can be worrying in ST131 clones given its virulent and pathogenic features [10]. To date, *fosA8* has been isolated from *E. coli* ST457 from Canada, and *E. coli* ST69 and ST410 from Switzerland, carried by IncN plasmids [11,12].

## Conclusion

The presence of the rare FosA8 in the healthcare setting poses an additional challenge for microbiological diagnosis and surveillance. So far, little is known on the route of transmission of FosA8-producing *E. coli* in Europe, thus requiring further investigation. Based on the global spread of acquired *fosA* genes, FOS surveillance programs, together with molecular approaches, would enable a better understanding of the prevalence of FOS resistance mechanisms. Moreover, insight into FosA enzyme circulation among Enterobacterales could help prevent FOS resistance and optimise therapeutic strategies.
